# Cold Regime Interannual Variability of Primary and Secondary Producer Community Composition in the Southeastern Bering Sea

**DOI:** 10.1371/journal.pone.0131246

**Published:** 2015-06-25

**Authors:** Beth A. Stauffer, Jennifer Miksis-Olds, Joaquim I. Goes

**Affiliations:** 1 Department of Biology and Paleo Environment, Lamont-Doherty Earth Observatory of Columbia University, Palisades, NY, 10964, United States of America; 2 Applied Research Laboratory, The Pennsylvania State University, State College, PA, 16803, United States of America; CSIR- National institute of oceanography, INDIA

## Abstract

Variability of hydrographic conditions and primary and secondary productivity between cold and warm climatic regimes in the Bering Sea has been the subject of much study in recent years, while interannual variability within a single regime and across multiple trophic levels has been less well-documented. Measurements from an instrumented mooring on the southeastern shelf of the Bering Sea were analyzed for the spring-to-summer transitions within the cold regime years of 2009–2012 to investigate the interannual variability of hydrographic conditions, primary producer biomass, and acoustically-derived secondary producer and consumer abundance and community structure. Hydrographic conditions in 2012 were significantly different than in 2009, 2010, and 2011, driven largely by increased ice extent and thickness, later ice retreat, and earlier stratification of the water column. Primary producer biomass was more tightly coupled to hydrographic conditions in 2012 than in 2009 or 2011, and shallow and mid-column phytoplankton blooms tended to occur independent of one another. There was a high degree of variability in the relationships between different classes of secondary producers and hydrographic conditions, evidence of significant intra-consumer interactions, and trade-offs between different consumer size classes in each year. Phytoplankton blooms stimulated different populations of secondary producers in each year, and summer consumer populations appeared to determine dominant populations in the subsequent spring. Overall, primary producers and secondary producers were more tightly coupled to each other and to hydrographic conditions in the coldest year compared to the warmer years. The highly variable nature of the interactions between the atmospherically-driven hydrographic environment, primary and secondary producers, and within food webs underscores the need to revisit how climatic regimes within the Bering Sea are defined and predicted to function given changing climate scenarios.

## Introduction

The Bering Sea ecosystem is of significant economic importance as the source for over 40% of finfish and shellfish landings, in biomass, in the United States [1]. These natural resources also provide subsistence for residents in rural communities, many of whom are Alaskan natives [1]. In addition the commercial fishing industry in the Bering Sea supports large businesses related to the acquisition, processing, and distribution of fish catch. The Bering Sea ecosystem is also ecologically significant as the home to at least 28 species of marine mammals, including pinnipeds and whales [2], and seabirds, which are supported by the extremely productive waters of the Bering Sea ‘Green Belt’[3].

In addition to the rich biological resources of the Bering Sea, Arctic and sub-Arctic ecosystems are often considered early indicators of climate change due to their tight coupling with atmospheric dynamics through the seasonal formation and retreat of sea ice. Signs of warming have become apparent in the northern Bering Sea and Arctic, with significant decreases observed in mean annual sea ice extent since 1979 and 1998 in the Chirikov Basin [4] and Arctic Ocean [5], respectively, and a 27% increase in annual mean open-water area since 1998 in the Arctic Ocean [6]. While secular trends in warming or sea ice cover have not yet been documented in the southern Bering Sea, several decades of research have revealed significant differences in environmental conditions and Bering Sea ecosystem function during warm and cold climatological regimes [1,7–9]. Shifts between these regimes, driven mainly by meteorological and atmospheric coupling with the Arctic [5,10], typically occur over 3–7 year cycles. Some of the major differences in Bering Sea ecosystem function during warm years (relative to cold) include: delayed timing of the spring phytoplankton bloom; shifts in zooplankton consumer populations with reduced populations of large copepods; and transport of the resulting biomass to pelagic rather than benthic fishery populations. These effects, among many others, were summarized by Hunt et al. (2002) in the Oscillating Control Hypothesis (OCH) to explain the ecological dynamics associated with each climatic regime [7]. The OCH identifies bottom-up or top-down control of fisheries in cold or warm regimes, respectively, related to the timing of the spring phytoplankton bloom, differential temperature-regulated zooplankton production, and impacts of secondary production on survival and recruitment of piscivorous fish. This hypothesis has been revised since its original inception to incorporate evidence for a lack of large zooplankton in warm years [1,8,11], despite increased primary production [12].

Many specific studies have examined the impacts of the oscillation between warm and cold regimes within the Bering Sea on food webs in the region. Recently, Sigler et al. (2014) suggested relatively stationary temporal dynamics between sea ice presence/retreat and timing of the spring bloom since the late 1990s [13]. Analysis of data from moorings in the southeastern Bering Sea predicted that spring blooms occur in April when ice is present at that time (e.g. in cold years) or in late May-early June when ice has already retreated, often followed by a fall phytoplankton bloom [13]. Somewhat conversely, the timing of the spring bloom and phytoplankton community composition in the southeastern Bering Sea has also been shown to vary significantly within a single climatic regime, with potential impacts on zooplankton consumers [14]. With a focus on consumers, Coyle and Pinchuk (2002) observed an apparent lack of significant interannual differences in euphausiid biomass on the inner shelf of the southeast Bering Sea from 1997 to 1999, suggesting that biomass of a single class of zooplankton is relatively invariant over small time-periods [15]. However, comparison of summer zooplankton abundances from 1999 to 2004 indicated a substantial shift from large to small copepod species correlated with increased water column stability and warming [16]. These studies illustrate the variability observed in investigations based on relatively limited trophic and/or temporal resolution. This lack of attention is not due to a lack of interest or effort, but rather is largely a result of technological limitations in present abilities to resolve complex community dynamics over time in a remote, ice-covered environment.

The current study investigated the potential for interannual variability within a single cold regime and its relation to the level of primary producers and community composition of primary and secondary producers. This work utilized unique data from mooring M2 in the southeastern Bering Sea including data on the physical water column, primary producer biomass, and acoustically-derived zooplankton/fish abundance and community composition estimates. The objective was to investigate to what degree interannual variability within a cold regime translates into significant differences in the timing and magnitude of phytoplankton blooms and trophic responses of secondary producers and consumers.

## Methods

### Mooring site and description

**Fig 1:**
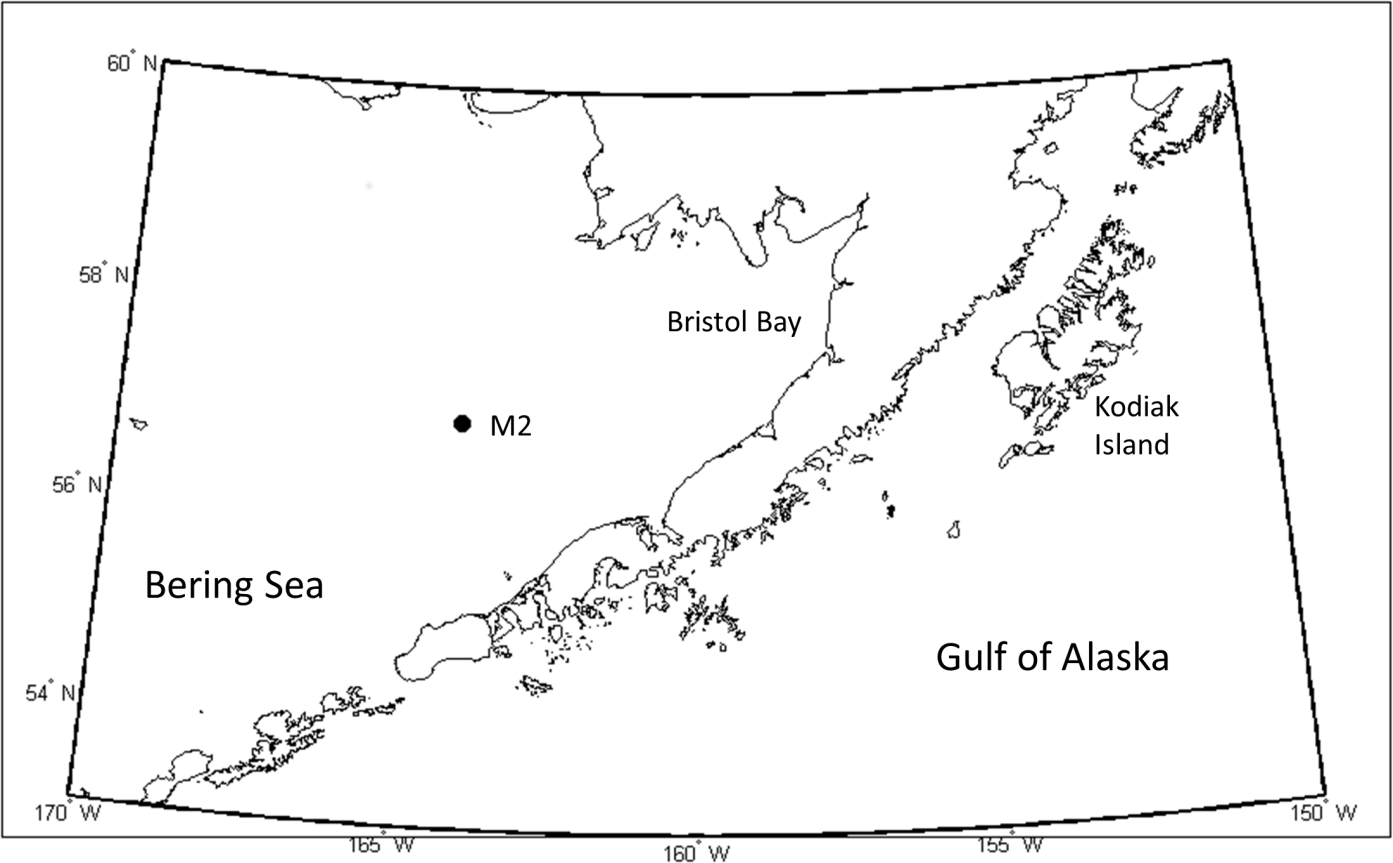
Map of the study area. Eastern Bering Sea and Gulf of Alaska, noting the location of the M2 mooring (circle), relevant seas and land areas.

Mooring Site M2 is located at 56.86° N 164.06° W along the 70 m isobath of the continental shelf in the southeastern Bering Sea (Fig 1). The M2 moorings have been maintained since 1995 as part of the Eco-FOCI project (http://www.ecofoci.noaa.gov), which has also maintained mooring sites to the south and north of M2 [13,17]. The M2 mooring is composed of two subsurface moorings (oceanographic and acoustic) separated by approximately a kilometer to minimize noise from the oceanographic mooring chain and sensors in the acoustic recordings. The moorings were deployed sub-surface, allowing them to persist through ice-covered winters, and were typically recovered and re-deployed in early spring (April/May) and fall (September/October) of each year. The oceanographic mooring was equipped with CTDs (Sea-Bird Electronics, SBE-37), temperature (Sea-Bird Electronics, SBE-39), and nitrate (Satlantic, MBAI-ISUS VI) sensors at shallow (< 20 m), mid-column (20–40 m) and deep (> 40 m) depths. Shallow and mid-column chlorophyll *a* (Chl *a*) fluorometers (WET Labs, ECO Fluorometer) were located at depths of 11 m and 32 m. The factory calibration was used to convert chlorophyll fluorescence to Chl *a*. These are only estimates based on fluorescence of Chl *a*, as direct chlorophyll samples were only taken at the site during deployments and recoveries. Data were collected at least hourly, and all instruments were calibrated prior to deployment. The data were processed according to manufacturers’ specifications. A low pass filter (35-hour Lanczos squared) was applied to each of the oceanographic series [18], and the series were averaged over 6 hour intervals.

Sensors on the acoustic mooring from September 2008—September 2009 consisted of an upward-looking 300 kHz RDI ADCP at 58 m, a three-frequency (125 kHz, 200 kHz, and 460 kHz) scientific echosounder system of Acoustic Water Column Profilers (AWCPs: ASL Environmental Sciences, Inc, British Columbia, Canada) at 65 m, and a Passive Aquatic Listener (PAL) recorder at 67 m. In September 2009, the mooring configuration changed to add an additional passive acoustic recorder at 66 m, which shifted the PAL to 65m, the AWCP system to 63 m, and the ADCP to 56 m. The AWCP system was mounted in an upward-looking direction 15° off vertical. The vertical offset eliminated interference from flotation and instruments in the mooring line directly above the active acoustic system. AWCPs monitor the presence and location of acoustic scatterers such as zooplankton and fish within the water column [19,20].

Hydrographic data were available from the M2 mooring from September 2008 until September 2012. Due to sensor malfunctions, a prolonged gap exists from September 2009 to May 2010 and, as a result, data from the 2010 spring-summer transition (March—August) were excluded from subsequent analyses comparing oceanographic conditions, primary producer and consumer abundance, and community composition. In addition, data from the deep fluorometer measuring Chl *a* were absent in March—April 2009 and after late September 2009. As a result, only surface and mid-column chlorophyll were utilized in the analyses. Data from the acoustic mooring were not available from November 2008 to April 2009 due to damage from a fishing vessel in October 2008; as a result, analyses of the 2009 spring-summer transition were limited to the data from May through August.

### Ice data

Data on daily mean ice cover (or percent cover in this specific region) and ice thickness were obtained from the images produced by the NOAA Ice Desk at the National Weather Service in Anchorage, Alaska. The images are based on multiple data sources, including satellite and ship-based observations, and are available at http://pafc.arh.noaa.gov/ice.php. Ice conditions surrounding the M2 mooring location were estimated within an approximately 20 km^2^ area around the mooring.

### Data processing

Temperature, salinity, and Chl *a* data were binned into daily mean values. Short gaps (< 2 weeks) in sensor data and a month-long gap in Chl *a* values in August 2009 were interpolated and extrapolated, respectively, using linear trends. Density anomaly (σ) was computed for shallow and deep depths using mooring temperature, salinity, and pressure data [21]. Stratification was estimated as the difference in density anomaly (Δσ) between shallow and deep depths [22]. The date of increased stratification was estimated as the date when Δσ > 0.8, a difference that corresponded to periods when Δσ was generally increasing in each spring-summer transition. Stratification was not computed for the incomplete hydrographic dataset in 2010.

Data on ice extent (% of area covered) and thickness (cm) were available every 2–3 days. During ice-free conditions (i.e. surrounding data indicated no ice), 1–2 day gaps in data were set to zero. During ice-covered conditions, missing data were interpolated daily using nearest-neighbor methods [23]. For each year, the date of ice retreat was identified as the date when ice extent was ≤ 15% for the last time that spring, consistent with the definition of ice retreat used by Sigler et al. [13]. Additionally, the date of < 50 cm ice thickness was identified in each year as the date when ice thickness measured 50 cm for the last time. This benchmark in ice thickness has been shown to have the potential for ecological significance given the rapid attenuation of light through sea ice < 50 cm thick compared to the relatively reduced attenuation through deeper levels of ice [24].

**Fig 2:**
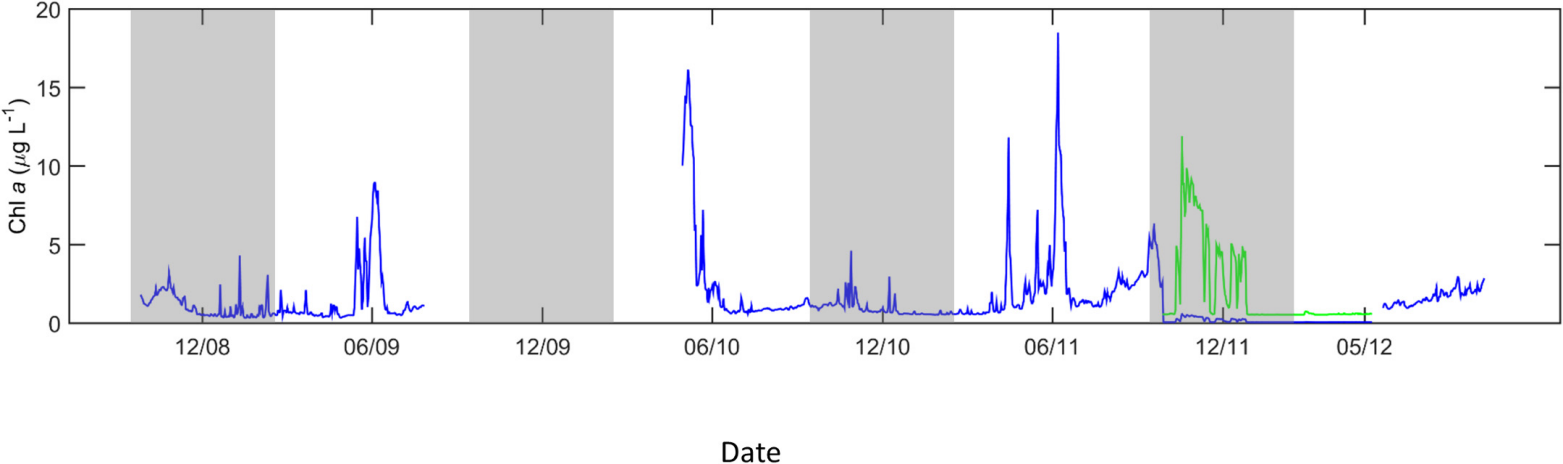
Chl *a* fluorescence correction. Uncorrected (blue) and mean- and standard deviation-corrected (green) shallow Chl *a* fluorescence data from the M2 mooring. Data from September 2011 to May 2012 were corrected due to an equipment exchange that resulted in abnormally low, unscaled values (see Methods for full details). Data from September to February in each year (grey boxes) were not included in statistical analyses in the current study.

Additional processing was required for the shallow Chl *a* data for the period September 2011—May 2012. The shallow fluorometer was replaced in Sept 2011 with a different instrument (WET Labs, WETStar), and the values decreased to < 0.1 μg Chl *a* L^-1^ for much of the subsequent deployment (Fig 2), despite ship-board measurements at M2 > 1.0 μg Chl *a* L^-1^ in early May 2012 [14]. Within these exceedingly low values were realistic patterns of variability, likely due to incorrect factory-calibrated scale factors. Such scale factors for optical instruments are susceptible to error due to a variety of issues including incorporation into a platform, vibrations during shipping, and physical damage [25,26]. Data from this period were therefore normalized using the period-specific mean (0.1 μg L^-1^) and standard deviation (0.1 μg L^-1^), and then re-computed using the mean (2.0 μg L^-1^) and standard deviation (2.6 μg L^-1^) from the preceding period, May 2010—September 2011. These processing steps resulted in a more realistic range (0.5–11.9 μg L^-1^) of Chl *a* during this period and better transition between data during this period and those preceding and following (Fig 2).

This correction was validated by comparison with data from a CTD-mounted fluorometer (WET Labs, WETStar) profiling at M2 at approximately noon local time on 8 May 2012 [14]. This fluorometer measured mean surface (< 5 m) Chl *a* concentrations of 2.1 μg L^-1^, approximately 3.4 times higher than that indicated by the reprocessed mooring data. Mid-column Chl *a*, which was not reprocessed, measured at this time by the mooring- and CTD-mounted fluorometers also differed by a factor of 2.4. Differences of this magnitude appeared to be within those attributable to differences in instrument calibration, small-scale spatial and temporal variability [27,28], and inherent differences in measurements of *in vivo* versus extracted pigment fluorescence [29] on which many fluorometer calibrations are based. We therefore concluded that the reprocessed mooring data provided a reasonable relative estimate of shallow Chl *a* concentrations during this time period. It should also be noted that all subsequent statistical analyses utilized only the mean-removed values from March through August in each year, thus closely reflecting the original data’s relative structure, excluding high Chl *a* values in Fall 2011, and minimizing influences of sensor drift or biofouling.

Acoustic backscatter magnitude and corresponding community composition estimates were assessed from daily measurements integrated over the entire water column from approximately 1 m above the AWCP transducer face to the surface. The surface was identified as 2 m below the visible surface line on echograms displayed in EchoView (Myriax, Tasmania) to minimize the contribution of surface bubbles in the biological analysis. The AWCP data were collected at 20 cm vertical resolution and processed in 5 m vertical depth bins. Mean volume backscatter coefficient (mean S_v_ in units m^2^ m^-3^) was calculated from integrations in 24 hour bins over each 5 m depth layer using EchoView software. Targets were then classified as to the likely source of the scattering based on differences in scattering amplitude between the three frequencies. Analyses using this dB-difference approach [30–32] are typically ground-truthed with information from net tows or video observations. Zooplankton net tows were conducted on mooring deployment and recovery cruises, as well as on separate research cruises in the area, with either a 25-cm diameter CalVET system (CalCOFI Vertical Egg Tow; [33]) or double-oblique tows of paired bongo frames at and around the moorings. Dominant species, species composition, and numerical density were identified from these samples, and this information was used to inform the size and animal group categorization of the acoustic data consistent with methods in Miksis-Olds et al. (2013).

Given the paucity of direct sampling of the water column under ice in this study, the dB-difference for a single scatterer type and an aggregation of scatterers of this type were considered to be identical given mono-specific scattering assemblages, despite differences in volume backscattering at each frequency. Theoretical scattering curves for four different size groups of scatterers were generated and dB-differences at the three acoustic frequencies used in this study were calculated. Scattering amplitudes (and the subsequent dB differences at 125, 200, and 460 kHz) were generated using a Stochastic Distorted Wave Born Approximation model [34] for the following scatterers: 1) small scatterers, such as copepods (lengths: 1–5 mm, “SmCrust”); 2) medium scatterers (lengths: 5–15 mm, “MdCrust”), which includes juvenile krill, chaetognaths, and amphipods; 3) large scatterers (lengths: 15–30 mm, “LgCrust”), such as adult euphausiids; 4) resonant scatterers; and 5) unknown (“Unclass”) [35]. The acoustic system was not able to detect the weak scattering strengths of scatterers less than approximately 5 mm in length unless they were present in extremely dense aggregations. Neritic copepod species typically found over the middle shelf (*Pseudocalanus* spp., *Acartia longiremis*, *Oithona* spp. and *Calanus*) are less than 5 mm [15,36] and comprised the small scatterer category. The resonant scatterer type represents an organism with a gas-inclusion such as a swim-bladdered fish or siphonophore which has a strong resonant peak in the scattering spectra [37]. Two theoretical resonant scattering spectra were generated: weak (< 3 dB increase in S_v_ at 200 kHz, “WkReson”) and strong (> 20 dB increase in S_v_ at 200 kHz, “StReson”). Aggregations were classified as belonging to one of the five categories (small, medium, or large scatterer; resonant; or unknown) by determining the shortest geometric distance between the three dB differences calculated for the aggregation and that of the theoretical scatterers. If the closest geometric distance was more than 12 dB (an arbitrarily-chosen value), then the aggregation was classified as unknown.

Short data gaps (1 and 6 days in 2011 and 2012, respectively) in acoustic scattering data occurred during time periods of mooring maintenance. Values during these gaps were estimated using linear interpolation of the gridded dataset. A larger data gap in the community composition exists from March-September 2010 due to a malfunction in the 460 kHz AWCP sensor. Three frequencies are required for the dB-differencing technique described above, so analysis was limited during this time period to the relative magnitude changes of S_v_.

### Statistical analyses

Environmental conditions during the 2009, 2010, 2011, and 2012 spring-summer transitions from March—August each year were estimated using parameters summarized in Table 1. Using Primer v.6, mean and maximum ice extent, ice thickness, mean and maximum shallow temperature, and salinity were normalized using mean and standard deviation from the March—August time period each year [38]. Dissimilarities in environmental conditions between years were calculated using Euclidean distance [38] and were used in hierarchical cluster analysis with similarity profile permutation tests (SIMPROF, Primer v.6) to examine statistical significance of dissimilarities among the years (p < 0.05). Nonmetric multidimensional scaling (MDS) was utilized to graphically depict the drivers of environmental conditions in each year, with results from the SIMPROF test outlining significantly different years.

**Table 1:** General environmental conditions during the spring-summer transitions in 2009, 2010, 2011, and 2012.

	2009	2010^†^	2011	2012
Max Ice (%)	85	85	80	85
Max Ice (cm)	100	80	61	120
Mean Ice (%)	13.9	24.0	17.6	35.3
Mean Ice (cm)	11.3	18.3	9.12	23.7
Day Ice Retreat	140	158	138	184
Day Ice < 50 cm	119	128	126	144
Stratification Day (Δσ)	169	—	187	122
Max Shallow Temperature (C)	10.1	8.36	10.4	8.94
Mean Shallow Temperature (C)	3.38	3.40	3.51	2.73
Max Shallow Salinity	31.9	31.8	31.9	31.8
Mean Shallow Salinity	31.6	31.7	31.5	31.5

Maximum (Max) and mean values were calculated using data from March through August in each year, except as indicated for 2010. Dates are presented as Julian days. Stratification Day (Δσ) was not calculated for 2010 due to discontinuities in the temperature and salinity data.

^†^ Shallow temperature and salinity data were available from 30 April to 5 August 2010.

To quantify the extent to which hydrographic and biological constituents measured on the M2 mooring varied together, cross-covariance (*cov*) of the mean-removed, processed data were computed using the ‘xcov’ function in Matlab (v. R2013a, Mathworks). The ‘coeff’ scaling option was employed to normalize *cov* and facilitate comparison across pairs of variables. As a result, maximum and minimum values of 1 and -1 were possible for *cov*, representing strong covariance of maxima with maxima (and minima with minima) or maxima with minima between two parameters, respectively. Time lags between signals (< 150 d) were identified from the maximum absolute values of *cov*, with positive values representing a lead of the first process and negative values indicating a lag [39]. In addition to interrogations of the strength of covariance for specific pair-wise comparisons, the mean of the highest absolute values of *cov* (|*cov*|) for each initial parameter (n = 28) were compared across years using a t-test or, when normality was not sufficient, a Mann-Whitney Rank Sum test (SigmaPlot v. 11.0, Systat Software).

### Ethics Statement

The acoustic sensors were integrated into the NOAA-deployed, observational moorings under a NOAA Request for Blanket Scientific Research Permit and did not require a specific permit for remote sensing.

## Results

### Spring-summer environmental conditions

**Fig 3:**
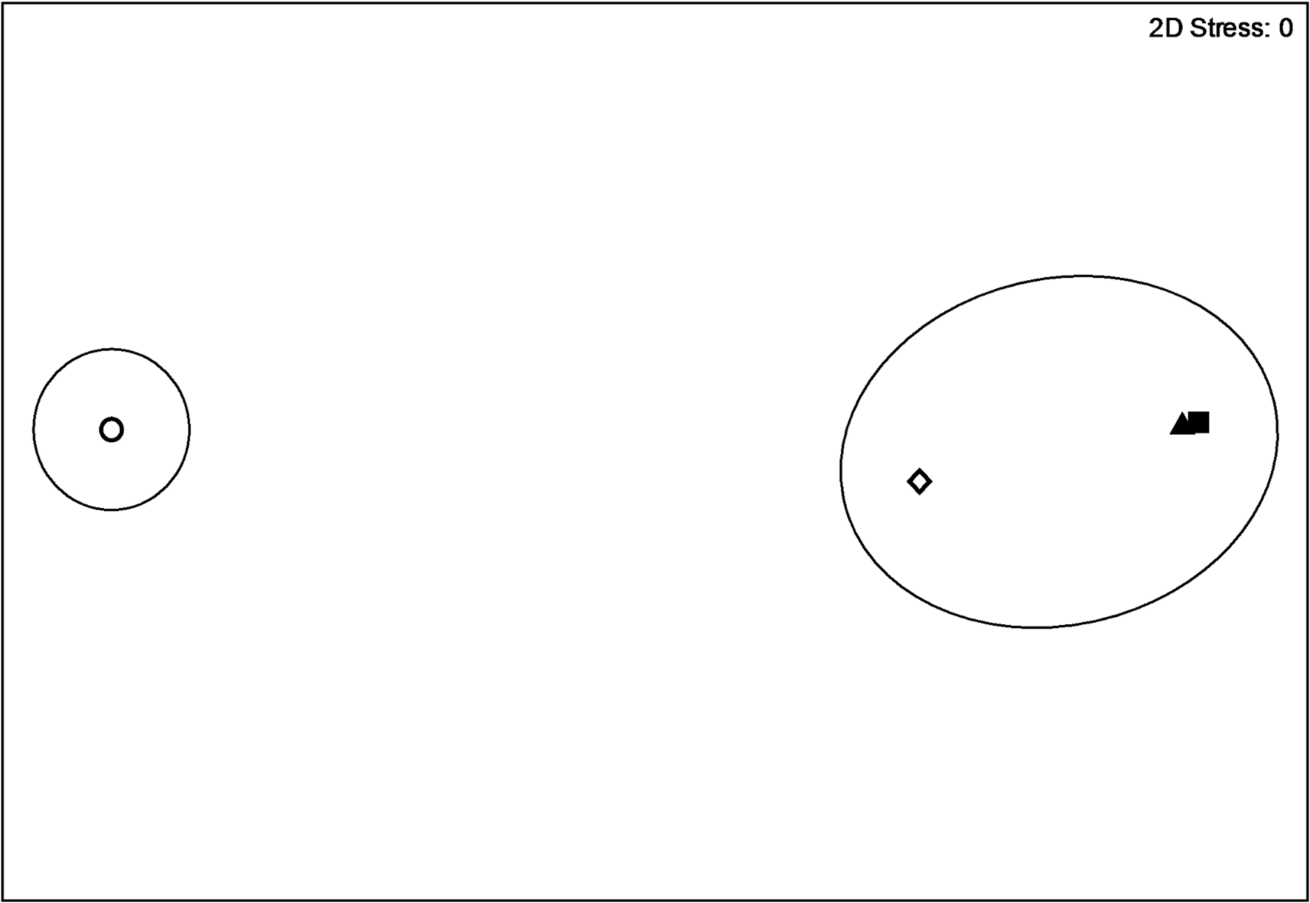
Non-metric multi-dimensional scaling of general environmental conditions at M2 mooring. Environmental conditions in 2009 (filled triangle), 2010 (open diamond), 2011 (filled square), and 2012 (open circle) based on the data from Table 1. Data were normalized to mean and standard deviation and Euclidean distance was used to calculate resemblance. Significant dissimilarities between environments in 2012 and the other three years (p < 0.05) are indicated by the black circles (Euclidean distance = 5) and are based on the permutation-based SIMPROF test (Primer v. 6). Axes are unit-less.

Taking into account the gaps in data, 3 full spring-summer transitions were recorded from the M2 mooring: 2009, 2011, and 2012. Data from 2010 were limited to late April through August and were only used to compare environmental conditions among the four years. Maximum ice extent was not considerably different among years, though mean ice extent and thickness were higher in 2012 than in 2009, 2010, or 2011 (Table 1). Additionally, ice retreat was delayed in 2012 by 44 and 46 days compared to 2009 and 2011, respectively, and by 26 days compared to 2010 (Table 1). In general, 2012 was colder with mean and maximum shallow temperatures of 2.7 and 8.9° C, respectively, and was characterized by slightly lower shallow salinity, likely due to the presence of sea ice later into the season (Table 1). Multivariate cluster and MDS analyses indicate that the spring-summer environment in 2012 was significantly different (p < 0.05) from the environment in the other three years which were largely similar to one another (Fig 3).

### Hydrography and primary producer biomass

#### 2009

**Fig 4:**
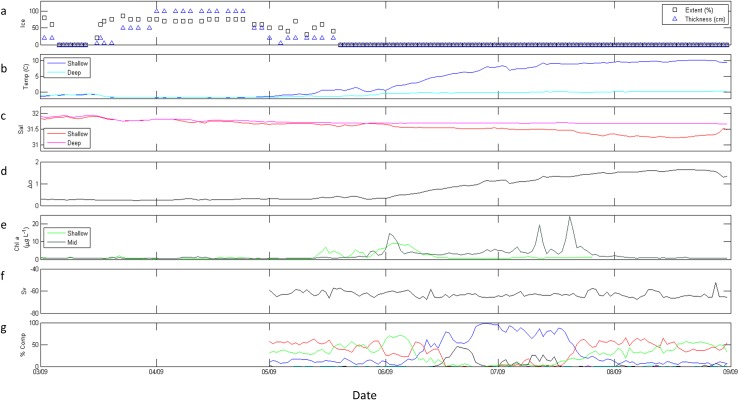
M2 data from March through August 2009. (a) ice extent (%, black boxes) and thickness (blue triangles); (b) shallow (dark blue) and deep (cyan) temperature (°C); (c) shallow (red) and deep (magenta) salinity; (d) stratification (Δσ); (e) shallow (light green) and mid-column (dark green) Chl *a* fluorescence (μg L^-1^); (f) total scattering volume (S_v_); (g) percent composition of classes of scatterers, including SmCrust (black), MdCrust (blue), LgCrust (red), WkReson (green), StReson (cyan), and Unclass (magenta). Note that scattering data was unavailable March—April 2009.

Spring 2009 (Fig 4) was physically distinct compared to Spring 2011 and 2012 in the occurrence of a significant mid-winter ice retreat over 7 days in March at M2 (Fig 4A). This retreat, and its effects on the ecosystem, was also observed at the Eco-FOCI M5 mooring to the northwest of M2 [35]. Surface temperature at M2 increased from a 7-day mean of -1.1 to -0.8° C during the retreat period, and decreased to -1.4° C once ice returned (Fig 4A). At M2, ice was present until mid-May, after which changes in temperature and salinity (Fig 4B and 4C) resulted in initial increase in stratification (Δσ) in late May (Fig 4D). Shallow Chl *a* began to increase while ice was still present, reaching a peak (9.0μg L^-1^) in early June 2009, two days after a mid-column Chl *a* bloom (14.7 μg L^-1^) and 15 days following ice retreat (Fig 4E). By mid-June, shallow and mid-column Chl *a* were consistently < 10.0 μg L^-1^ until a mid-column, bi-modal bloom began in mid-July, peaking at 19.4 and 24.4 μg L^-1^ on 12 and 20 July 2009, respectively. Shallow Chl *a* did not exceed 1.5 μg L^-1^ after the initial May-June bloom.

#### 2010

**Fig 5:**
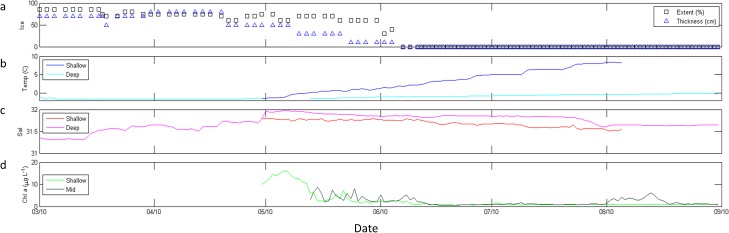
M2 data from March through August 2010. (a) ice extent (%, black boxes) and thickness (blue triangles); (b) shallow (dark blue) and deep (cyan) temperature (° C); (c) shallow (red) and deep (magenta) salinity; and (d) shallow (light green) and mid-column (dark green) Chl *a* fluorescence (μg L^-1^).

Ice was present until early June 2010 (Fig 5A), and shallow temperature began increasing in May (Fig 5B) concomitant with an increase salinity at depth (Fig 5C). Evidence for a shallow phytoplankton bloom in early May was indicated by an increase in Chl *a* to 16.1 μg L^-1^ (Fig 5D). Mid-column Chl *a* showed two apparent periods of increased biomass with maximum concentrations of 8.8 μg L^-1^ in late May 2010 (Fig 5D).

#### 2011

**Fig 6:**
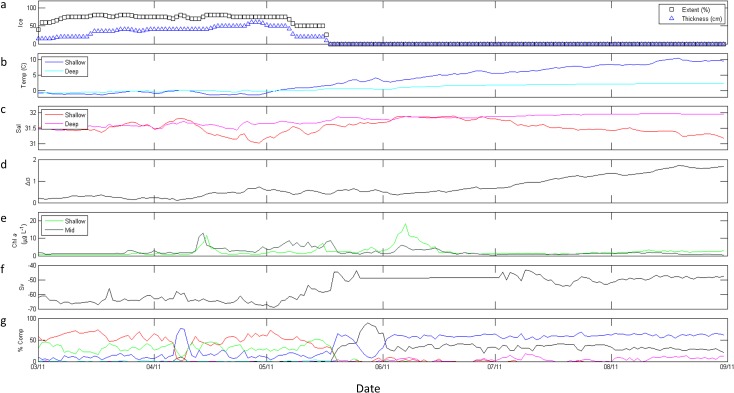
M2 data from March through August 2011. (a) ice extent (%, black boxes) and thickness (blue triangles); (b) shallow (dark blue) and deep (cyan) temperature (° C); (c) shallow (red) and deep (magenta) salinity; (d) stratification (Δσ); (e) shallow (light green) and mid-column (dark green) Chl *a* fluorescence (μg L^-1^); (f) total scattering volume (S_v_); (g) percent composition of classes of scatterers, including SmCrust (black), MdCrust (blue), LgCrust (red), WkReson (green), StReson (cyan), and Unclass (magenta).

Ice was present until mid-May in 2011 (Fig 6A) as well, following a 4-day mid-winter ice retreat in February. This mid-winter ice retreat was accompanied by slight surface warming to a 7-day mean temperature of -0.7° C during the retreat (data not shown), followed by cooling to -0.8° C upon the return of ice. An initial increase in stratification began in early April 2011 (Fig 6D), likely a result of decreased salinity at shallow depths during that time (Fig 6C). However, stratification did not increase again until early June (Fig 6D) with increasing and decreasing temperature (Fig 6B) and salinity (Fig 6C), respectively, at shallow depths. Shallow and mid-column Chl *a* began to increase in late March and reached local maxima (11.8 μg L^-1^ and 13.2 μg L^-1^, respectively) within one day of each other in mid-April, approximately one month before ice retreat. (Fig 6E). Chl *a* decreased following these peaks until mid-May when smaller-scale increases in shallow Chl *a* (7.2 μg L^-1^) and mid-column Chl *a* (8.9 μg L^-1^) were observed, still 34 days before ice retreat. Shallow Chl *a* reached 18.5 μg L^-1^ in early June 2011, 20 days after ice retreat, while a later increase in mid-column Chl *a* to 6.0 μg L^-1^ in early June 2011 was less distinct.

#### 2012

**Fig 7:**
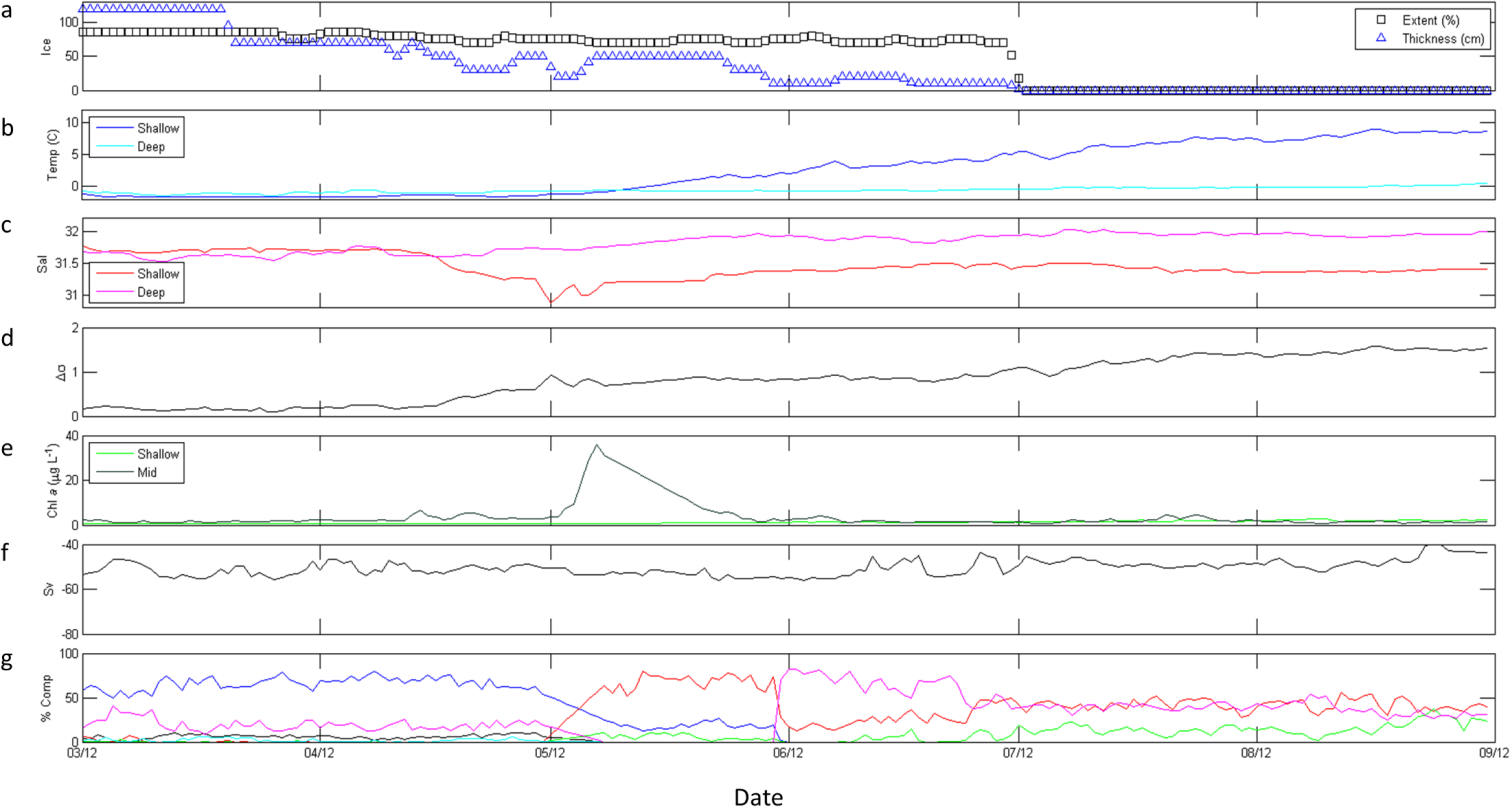
M2 data from March through August 2012. (a) ice extent (%, black boxes) and thickness (blue triangles); (b) shallow (dark blue) and deep (cyan) temperature (° C); (c) shallow (red) and deep (magenta) salinity; (d) stratification (Δσ); (e) shallow (light green) and mid-column (dark green) Chl *a* fluorescence (μg L^-1^); (f) total scattering volume (S_v_); (g) percent composition of classes of scatterers, including SmCrust (black), MdCrust (blue), LgCrust (red), WkReson (green), StReson (cyan), and Unclass (magenta). Note the different scale for Chl *a* (e), with a maximum of 40 μg L^-1^.

Ice around M2 was generally more abundant (mean extent > 35%) and thicker (mean thickness > 23 cm) in 2012 compared to the other 3 years (Table 1). Furthermore, no mid-winter retreat was observed in 2012 (Fig 7A), and the date of ice retreat was 44–46 days later than in the previous years. Similar to conditions in 2011, stratification in 2012 increased in two stages (Fig 7D): the first increase appeared driven mainly by salinity decreases at shallow depths (Fig 7C) and the second by increasing shallow temperature (Fig 7B). Shallow Chl *a* remained low throughout the March-August dataset (< 3.0 μg L^-1^), reaching a maximum in early August, 38 days after ice retreat (Fig 7E). Mid-column Chl *a* initially increased to 6.7 μg L^-1^ by mid-April, more than two months prior to ice retreat, and a maximum of 36.0 μg L^-1^ was reached in early May 2012 (Fig 7E), 23 days following the initial increase in biomass and still 56 days before ice retreat.

### Secondary consumer biomass and composition

In all three years, tradeoffs were apparent in relative abundance estimates of small and medium zooplankton scatterers and of medium and large zooplankton scatterers (Figs 4, 6, and 7). Large-scale variability of total scattering was minimal in 2009 (Fig 4) and 2012 (Fig 7F); however S_v_ showed a step-wise increase in May 2011 (Fig 6F) and higher values of S_v_ continued into 2012 (Fig 7F). Scattering communities in 2009 (Fig 4G) and 2011 (Fig 6G) were dominated by large zooplankton scatterers and weak resonant scatterers until approximately June in each year. Unclassified scatterers did not significantly contribute to the overall community in these years. In contrast, medium zooplankton scatterers dominated the community in spring 2012 until May when large zooplankton scatterers gained dominance followed by unclassified scatterers, which comprised > 80% of the community in June (Fig 7G). Additionally, small zooplankton scatterers did not make up more than 10% of the total scattering at any time in 2012 (Fig 7G) despite comprising ≥ 50% of the community in 2009 (Fig 4G) and 2011 (Fig 6G) once ice had retreated in those years. Finally, large zooplankton scatterers increased in response to maximum mid-column Chl *a* concentrations in May 2012 (Fig 7G), rather than the small or medium zooplankton scatterers which were responsive within 2 weeks to these maxima in 2009 (Fig 4G) and 2011 (Fig 6G).

### Cross-covariance across years

**Fig 8:**
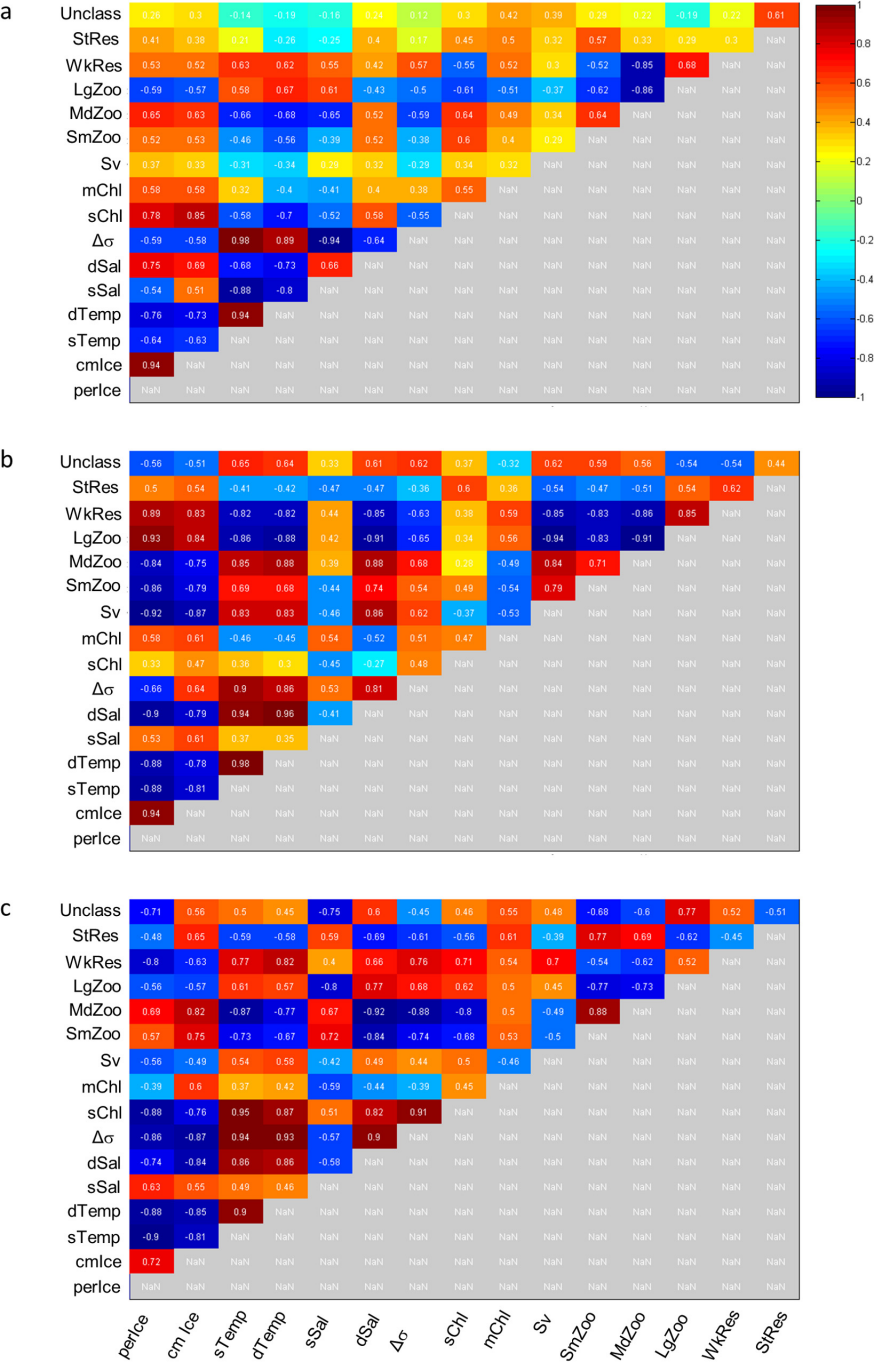
Heat map of *cov* values for pair-wise covariance analyses. (a) 2009, (b) 2011, and (c) 2012 covariance results. The strength of covariance is indicated by colors, with strong positive values of *cov* indicated by dark red and strong negative values of *cov* indicated by dark blue (see color bar). Weak levels of covariance are indicated by light orange to light blue colors. Values for *cov* are indicated within each box, and greyed-out boxes containing “NaN” indicate the converse comparisons which were not carried out.

The magnitude of pair-wise cross-covariance (*cov*) between the hydrographic and biological variables were calculated and showed significant variability between the three years (Fig 8). Absolute values of *cov* were generally lower (Fig 8, light-yellow to light-blue colors) in 2009 and 2011 than in 2012. When the top two absolute values of *cov* for each pair-wise comparison were considered (Table 2), the mean of the overall strength of covariance was significantly higher (p < 0.05; t-test, df = 54) in 2012 (mean |*cov*| = 0.782) compared to 2009 (mean |*cov*| = 0.682) or 2011 (mean |*cov*| = 0.769). Mean absolute values of *cov* in 2009 and 2011 were not statistically different from each other (p > 0.05; t-test, df = 54).

**Table 2:** Summary of top two *cov* values for each pair-wise cross-covariance analysis.

	2009			2011			2012		
	cov	Variable	Lag	cov	Variable	Lag	cov	Variable	Lag
**ShTemp**	-0.642	% Ice	0	-0.880	%Ice	0	-0.898	% Ice	0
	-0.627	cm Ice	0	-0.812	cmIce	0	-0.815	cmIce	0
**DpTemp**	0.940	ShTemp	0	0.980	ShTemp	0	0.902	ShTemp	0
	-0.762	% Ice	0	-0.877	%Ice	0	-0.876	%Ice	0
**ShSal**	-0.884	ShTemp		0.609	cmIce	61	0.631	%Ice	-74
	-0.804	DpTemp	0	0.375	ShTemp	-48	0.489	ShTemp	-137
**DpSal**	0.753	%Ice	0	0.962	DpTemp	0	0.864	ShTemp	0
	-0.732	DpTemp	0	0.938	ShTemp	0	0.861	DpTemp	0
	0.980	ShTemp	0	0.897	ShTemp	0	0.945	ShTemp	0
	-0.942	ShSal	0	0.859	DpTemp	0	0.931	DpTemp	0
**ShChl**	0.846	cmIce	23	0.476	Δσ	-73	0.950	ShTemp	0
	-0.697	DpTemp	14	0.470	cmIce	39	0.905	Δσ	0
**MidChl**	0.583	%Ice		0.608	cmIce	12	0.604	cmIce	63
	0.553	ShChl	43	0.538	ShSal	-44	-0.592	ShSal	5
**S**_**v**_	0.366	%Ice	6	-0.923	%Ice	0	0.584	DpTemp	0
	-0.339	DpTemp	6	0.862	DpSal	0	-0.558	%Ice	0
**SmCrust**	0.603	ShChl	17	-0.856	%Ice	2	-0.842	DpSal	2
	-0.555	DpTemp	47	0.736	DpSal	2	0.750	cmIce	10
**MdCrust**	-0.684	DpTemp	47	0.883	DpTemp	0	-0.923	DpSal	0
	-0.658	ShTemp	42	0.882	DpSal	0	0.884	SmCrust	0
**LgCrust**	-0.857	MdCrust	0	0.929	%Ice	1	-0.799	ShSal	12
	0.666	DpTemp	47	-0.912	MdCrust	0	-0.767	SmCrust	0
**WkReson**	-0.852	MdCrust	0	0.885	%Ice	2	0.816	DpTemp	0
	0.682	LgCrust	8	-0.859	MdCrust	0	-0.797	%Ice	0
**StReson**	0.566	SmCrust	51	0.625	WkRes	0	0.765	SmCrust	1
	0.499	MidChl	21	0.598	ShChl	-25	0.690	MdCrust	-1
**Unclass**	0.611	StRes	-17	0.651	ShTemp	0	0.772	LgCrust	22
	0.419	MidChl	5	0.642	DpTemp	0	-0.751	Surf Sal	39

Initial variables are listed in columns and include shallow temperature (ShTemp), deep temperature (DpTemp), shallow salinity (ShSal), deep salinity (DpSal), stratification (Δσ), shallow Chl *a* fluorescence (ShChl), mid-column Chl *a* fluorescence (MidChl), total scattering (S_v_), small crustaceans (SmCrust), medium crustatceans (MdCrust), large crustaceans (LgCrust), weak resonant scatterers (WkReson), strong resonant scatterers (StReson), unclassified scatterers (Unclass). Ice extent (% Ice) and thickness (cmIce) were also used. Comparison variables yielding the highest absolute cov values are listed under “Variable” in each year. Lags are indicated in days, Positive lag values indicate a lag of the comparison variable (column) relative to the initial variable (row); negative values indicate a lead of the comparison value (column) relative to the initial variable (row).

In all three years, temperature and salinity tended to, unsurprisingly, co-vary with ice conditions, each other, and stratification (Fig 8), often with zero lag (Table 2). Shallow Chl *a* co-varied most strongly with ice thickness in 2009, stratification in 2011, and shallow temperature in 2012 (Table 2). While these three parameters were all highly covariant in all years, as mentioned above, the variability in strength of *cov* is noteworthy. Mid-column Chl *a* was less strongly related to other measured parameters in 2009 and 2012, co-varying most strongly with ice conditions in both years (|*cov*| ≤ 0.604; Table 2). In 2011, however, mid-column Chl *a* co-varied more strongly with ice thickness (*cov* = 0.608) than shallow Chl *a* did with stratification that year (Table 2). Interestingly, mid-column and shallow Chl *a* co-varied only slightly with each other in any of the years (|*cov*| ≤ 0.553; Fig 8).

Total scattering did not strongly co-vary with any measured parameters in 2009 (|*cov*| ≤ 0.366), while S_v_ showed a strong, negative relationship with ice extent in 2011 (*cov* = -0.923) and moderate covariance with deep temperature and ice thickness in 2012 (Table 2). Overall covariance between the relative abundance of the different classes of scatterers and the measured hydrographic parameters was significantly weaker (p < 0.05; Mann-Whitney Rank Sum test) in 2009 (mean |*cov*| = 0.378) than in 2011 (mean |*cov*| = 0.531) or 2012 (mean |*cov*| = 0.605; Fig 8), suggesting a potential decoupling of environmental conditions and consumer populations during the warmer years in this dataset.

Within the scattering communities, small zooplankton scatterers co-varied most strongly with shallow Chl *a*, ice extent, and deep salinity in 2009, 2011, and 2012, respectively (Table 2), while medium zooplankton scatterers showed the strongest degree of covariation with deep temperature in 2009 and 2011, and deep salinity and, to a lesser extent, shallow Chl *a* (Fig 8), in 2012 (Table 2). The relative abundance of large zooplankton scatterers co-varied most strongly with the hydrographic parameters deep temperature, ice extent, and shallow salinity in 2009, 2011, and 2012, respectively (Table 2). Weak resonant scatterers were strongly covariant with ice extent and deep temperature in 2011 and 2012, respectively (Table 2), and with other scatterers, which is discussed below. Finally, strong resonant scatterers and unclassified scatterers co-varied only moderately with mid-column and shallow Chl *a* and temperature in 2011 and 2009 (Table 2). In general, shallow Chl *a* co-varied more strongly with zooplankton and weak resonant scatterers in 2012 (|*cov*| range: 0.616–0.804) than in 2009 (|*cov*| range: 0.516–0.635) or 2011 (|*cov*| range: 0.267–0.490; Fig 8), suggesting tighter coupling of primary production and consumer populations in the coldest year in the dataset.

Interactions among secondary consumers reveal several interesting patterns. Medium zooplankton scatterers positively co-varied with small zooplankton scatterers in all three years (*cov* range: 0.640–0.880). The strongest covariance was observed in 2012, and time lags of 6, 11, and 0 days in 2009, 2011, and 2012, respectively, were observed (Fig 8). Conversely, large zooplankton scatterers and weak resonant scatterers negatively co-varied with both small and medium zooplankton scatterers in all three years, with the strongest negative covariances observed in 2011 and time lags ≤ 1 day in all years (Fig 8). Strong resonant and unclassified scatterer covariance with other scatterers was generally variable in all years and only slightly stronger in 2012 (|*cov*| ≤ 0.770) than in 2009 or 2011 (|*cov*| ≤ 0.610) in 2009 and 2011 (Fig 8). Finally, time lags of strong resonant and unclassified scatterers with other consumer abundances were highly variable in all three years, ranging from 1 day (2011, 2012) to 51 days (2009) between strong resonant with small zooplankton scatterers (Fig 8).

## Discussion

### Cold regime phytoplankton blooms

From 2009 to 2012, a series of years classified as part of a “cold regime” [1], significant variability has been observed in this and other studies (e.g. [13]) in the timing of phytoplankton blooms relative to ice retreat. Within the cold years included in this study, overall conditions in 2009 and 2011 were very different from those in 2012, driven largely by differences in ice extent, ice thickness and dates of ice retreat and onset of stratification. Our data also suggest that predictions for when blooms will occur may not be as consistent as previously thought (e.g. [13]). Ice was still present in April in all of the years included in the current study (2009–2012), which according to Sigler et al. (2014) should lead to spring bloom formation also in April in those years. When treated as a local maximum between the months of March and August in the present dataset, however, the spring bloom only occurred in April in 2011, while significant blooms in 2009 and 2012 did not occur until May. These results suggest that bloom occurrence cannot be predicted based solely on the presence and/or extent of sea ice in cold regimes.

Analyses point to differing levels of covariance in phytoplankton biomass and hydrographic conditions during the spring-summer transitions in 2009, 2011, and 2012. In all years, variability in shallow and mid-column Chl *a* fluorescence was related to ice, temperature, density, and/or salinity, indicating the strong role played by sea ice dynamics in this environment, including the related formation of the cold pool [9,40]. The strength of covariance between phytoplankton biomass (measured as Chl *a* fluorescence) and hydrography varied across years, however, with significantly stronger relationships between hydrographic parameters and Chl *a* in 2012, the coldest and most environmentally distinct of the three years. These strong relationships suggest a tight coupling of hydrography and primary producer biomass in this colder year that was characterized by extensive, thick ice, consistently cold waters at depth, and a single, large phytoplankton bloom, compared to weaker coupling in the relatively warmer years of 2009 and 2011.

It is also noteworthy that shallow and mid-column Chl *a* did not strongly co-vary with each other in any of the years studied. This may be the result of both co-occurrence (e.g. June 2009 blooms) and/or independence (e.g. July 2009 bloom at depth) in depth-resolved bloom dynamics in both years, which would dampen the perceived strength of any potential covariance. These seemingly decoupled bloom dynamics also suggest the potential for development of depth-specific phytoplankton blooms independent of one another which may, in turn, stimulate different consumer populations and support primarily benthic (mid-column) or pelagic (shallow) food webs [12]. It is also possible that the mooring fluorometers were not at the depths required to resolve bloom dynamics (e.g. sinking) along this vertical dimension. However, the fact that depth-discrete blooms occurred more often following an increase in stratification (rather than before), suggests a physical mechanism to these depth-resolved dynamics and does not necessarily support sinking of surface populations to depth. Finally, this apparent lack of covariance in Chl *a* across depths supports the continued and expanded use of tools and approaches for resolving spatially- and depth-distinct phytoplankton communities in the field in addition to the largely surface-focused data provided through satellite-based remote sensing.

Furthermore, the composition of phytoplankton bloom communities at any depth is an equally important factor, with the timing and magnitude of blooms, in determining the food web impacts on changes to primary production in this ecosystem. Many consumers selectively feed within optimal size ranges for prey, and groups of phytoplankton vary in nutritional value, production of chemical defensive compounds, and employment of grazing-deterrent physiological strategies [41–43]. It is therefore important to not only quantify the amount of phytoplankton biomass in the water column and the timing of the spring bloom, but to also characterize the size distribution and composition of communities of primary producers.

Stauffer et al. (2014) documented the phytoplankton community composition during the 2011 and 2012 spring blooms at M2 captured in this dataset. Unfortunately, cruise-based data on phytoplankton community structure at M2 were unavailable for the early June 2009 or May 2010 spring blooms. The phytoplankton community in 2011 was sampled on 18 May, around the time of the broad mid-column Chl *a* peak (Fig 5E), and was dominated by microplankton (20–200 μm) at the surface and both micro- and picoplankton (< 3 μm) at 20 m [14]. In 2012, the M2 phytoplankton community was sampled on 8 May 2012, concomitant with the mid-column Chl *a* peak (Fig 6E) and was again dominated by microplankton at all depths but with a significant contribution from nanoplankton (3–20 μm) at 20 m [14].

Differences in size-fractionated Chl *a* in 2011 and 2012 were paralleled by significant differences in composition of the phytoplankton communities at M2. These differences were likely driven by a shift in dominance of the spring communities from small Prymnesiophytes in the genus *Phaeocystis* sp. in 2011 to larger diatoms (e.g. *Thalassiosira* sp.) in 2012 [14]. Prymnesiophytes are small cells with a cosmopolitan distribution and are capable of forming large, mucoid colonies. *Phaeocystis* spp. are considered an inferior copepod food source (compared to diatoms, e.g. [11]) due to their small individual and large colony sizes and potentially poor nutritional value given their low concentration of polyunsaturated fatty acids [44]. A diet based on *Phaeocystsis* sp. can result in lower grazing rates [44] and reduced fecundity [43] of copepod consumers. It is therefore likely that, in addition to differences documented in this study in magnitude and timing of the spring blooms at M2 in 2011 and 2012, the size distribution and composition of the phytoplankton communities in these years were capable of stimulating different levels and/or groups of consumers. Tools including but not limited to ship-based sampling, deployment of fixed-site moorings, and mobile sensing platforms are required to resolve these significant differences with depth with important trophic implications.

### Cold regime consumer communities

Consumer communities were resolved from distinct patterns of acoustic scattering (described in the Methods section), and showed differing responses to environmental conditions and primary producers in 2009, 2011, and 2012. In 2009 and 2011, initial consumer communities were dominated by large zooplankton scatterers (e.g. euphausiids) and weak resonant scatterers (e.g. fishes), while the initial 2012 community was dominated by medium-sized scatterers indicative of crustaceans. In the years for which we have continuous data (2011, 2012), these initial communities appeared to reflect the dominant communities in August of the prior year (2010, 2011).

Initial primary production stimulated different classes of secondary consumers in each year. In 2009 initial primary production primarily stimulated fishes (resonant scatterers), while small and medium zooplankton scatterers appeared to benefit from phytoplankton blooms in early May once the ice had retreated. The higher magnitude, late summer phytoplankton bloom stimulated large zooplankton scatterers and fishes in 2009, potentially via longer food webs (e.g. microzooplankton and small zooplankton), and these trophic interactions appeared to persist into fall. In contrast, medium zooplankton scatterer biomass increased in response to initial primary production in 2011 while sea ice was still present. The lower magnitude, prolonged bloom in May 2011 seemed to favor, in sequence, medium and then small sized zooplankton scatterers, once the ice had retreated, and medium scatterers appeared to persist throughout the remainder of 2011 and into the spring of 2012. Dominance of small and medium sized zooplankton scatterers in the late spring/early summer of 2011 is also potentially related to dominance of the May phytoplankton bloom at M2 by *Phaeocystis* spp. [14], a poor food source for large zooplankton, as discussed above. Interestingly, the subsequent bloom in June 2011 did not appear to stimulate any of the classified scatterers. In 2012, the sequence was greatly simplified, with the early May spring bloom, dominated primarily by diatoms [14], stimulating large zooplankton scatterers, followed by unclassified scatterers approximately one month later.

Small zooplankton scatterers, e.g. pelagic copepods < 5 mm in length, were largely absent in 2012, but did increase in abundance following spring blooms in 2009 & 2011 once ice had retreated, consistent with theories of metabolic (e.g. temperature) control of small zooplankton growth [45]. Given the strong, negative covariance between small zooplankton and ice extent or thickness in 2011, it is likely that the low abundance of small sized zooplankton in the cold year of 2012 is attributable to the presence of ice and colder temperatures until late June. This inverse relationship between small zooplankton abundance and ice presence is corroborated by the strong, negative covariance between these consumers and deep salinity, and may be indicative of a persistent, salty cold pool that forms during cold years in the Bering Sea [1]. Further evidence for a cold pool influencing M2 in 2012 includes strong stratification and consistently cold deep waters. These findings are consistent with previously documented patterns of increased abundance of smaller copepod species (e.g. *Pseudocalanus* spp., *Oithona* spp.) in warmer years [16,46], predominantly larger copepod species (e.g. *Calanus* spp.) in colder years [16,47], and increased success of larger zooplankton in years associated with cold pool development through spatial mismatch between large zooplankton and their predators [48]. Together, these results suggest that both delayed ice retreat and reduced consumption of these large zooplankton may have resulted in their survival and subsequent success into summer that year.

### Intra-consumer interactions

In all three years, a trade-off was apparent between small/medium- and large-sized zooplankton and fishes; however, the timing and dynamics of the transition in dominance among these groups varied across the years. In 2009, this trade-off was observed as a transition from large zooplankton and fish dominance early in the spring to small- and medium scatterer dominance from mid-June to mid-July. Large sized zooplankton and fishes returned to dominance in late July, however, likely a result of feeding on the smaller consumers. In 2011, the shift from initial large zooplankton and fish dominance to small and medium scatterer dominance occurred earlier, and this latter population of consumers remained dominant through August. In 2012, however, the transition was in opposition to that observed in the previous years: from medium scatterers to large zooplankton scatterers and, to a lesser extent, fishes, concomitant with the mid-column phytoplankton bloom in May. A subsequent transition to dominance of unclassified scatterers is unique to 2012 and underscores the need for further development of techniques to facilitate study of highly dynamic, poorly-characterized consumer populations.

This trade-off between smaller and larger size classes of consumers in each year is likely indicative of trophic interactions within the zooplankton and consumer communities. Chaetognaths (included within the medium-sized scatterer class) are considered abundant carnivores feeding mainly on small (e.g. *Pseudocalanus* spp.) and large (e.g. *Calanus* spp.) copepods in the Bering Sea [49]. Miksis-Olds et al. (2013) verified the contribution of chaetognaths to high abundance of medium zooplankton scatterers in 2009 using net samples [35]. This predator-prey relationship potentially explains the trends documented in the current study in which small and medium scattering groups varied in opposition to each other over periods of days in 2009 and 2011. This trophic relationship may also provide insights into why, in the absence of significant small copepod abundances in 2012, the population of medium, likely carnivorous, scatterers declined in early May despite a significant phytoplankton bloom occurring at that time.

Similarly, the diets of large zooplankton scatterers (e.g. euphausiids) include significant contributions from heterotrophic prey (e.g. copepods) in the western Pacific [50] and Southern Ocean [51]. Euphausiids are thought to remain abundant in the Bering Sea throughout the summer, fall and winter [51] and to show less variability between successive summers than copepods [15], patterns that are reflected in our observations from 2009 and 2012. Therefore, the decline in abundance of large scatterers such as euphausiids in the early summer of 2011 can be considered atypical, especially given the prolonged increase in primary production that year that stimulated production of smaller scattering classes of zooplankton. In addition, the diets of fishes, including juvenile and adult stages of economically important pollock in the Bering Sea, typically are dominated by contributions from medium to larger crustaceans, including copepods and euphausiids [52]. It is therefore reasonable to hypothesize that the simultaneous decline in acoustically-measured fish and euphausiid relative abundances in May 2011 was the result of 1) cascading effects of the dominance of an inferior phytoplankton food source (*Phaeocystis* spp.) in the community, which stimulated small and medium scatterer biomass, an inferior food source for higher trophic levels; and/or 2) top-down control of these populations, possibly by the unclassified scatterers or other consumers (e.g. marine mammals, fishing pressure) given the relative abundances of smaller copepod prey.

### Conclusions

A high degree of interannual variability was observed in the populations of primary and secondary producers and consumers within an individual “cold” climatic regime in the southeastern Bering Sea. The trophic interactions observed in the current study reflect a highly dynamic food web with many dimensions of production, predation, competition, and control that appear to be more tightly coupled to each other and environmental conditions in cold years than in relatively warmer years. The variable nature of the interactions between the environment, primary and secondary producers, and within food webs underscores the need for integrated food web investigations over multiple years both within and between climatic regimes. Improved resolution must include technological advancement in our abilities to remotely characterize phytoplankton size- and community structure and bloom dynamics at depth, as well as more sustained investigations of the interactions within communities of secondary producers. More specifically, the short temporal scales over which many of the observed trophic interactions occurred suggests an increased need for sustained *in situ* investigations of secondary production spanning entire seasons. Improved resolution of these complex community dynamics will facilitate more inclusive modeling of the Bering Sea ecosystem and contribute to our understanding of how differences between years, not necessarily representative of huge climatological shifts, can nevertheless have great ecological significance.

## Supporting Information

S1 Table Hydrographic and acoustic data from M2 Mooring. Hydrographic data (percent ice, cm ice, shallow and deep temperature, shallow and deep salinity, delta density, shallow and mid-column chlorophyll fluorescence) and acoustic data (total scattering) and relative abundance (small crustaceans, medium crustaceans, large crustaceans, weak resonators, strong resonators, unclassified scatterers) from 2009, 2010, 2011, and 2012 at the M2 mooring.
(XLS)

## References

[1] Stabeno PJ, Kachel NB, Moore SE, Napp JM, Sigler M, Yamaguchi A, et al Comparison of warm and cold years on the southeastern Bering Sea shelf and some implications for the ecosystem. Deep Sea Research Part II: Topical Studies in Oceanography. 2012;65–70(0):31–45. 10.1016/j.dsr2.2012.02.020.

[2] Perez MA, McAlister WB. Estimates of food consumption by marine mammals in the Eastern Bering Sea. US Dep Commer, NOAA Tech Memo NMFS-AFSC-141993.

[3] Okkonen SR, Schmidt GM, Cokelet ED, Stabeno PJ. Satellite and hydrographic observations of the Bering Sea ‘Green Belt’. Deep Sea Research II. 2004;51:1033–51.

[4] Brown ZW, van Dijken GL, Arrigo KR. A reassessment of primary production and environmental change in the Bering Sea. Journal of Geophysical Research-Oceans. 2011;116 10.1029/2010jc006766 .

[5] Brown ZW, Arrigo KR. Contrasting trends in sea ice and primary production in the Bering Sea and Arctic Ocean. Ices Journal of Marine Science. 2012;69(7):1180–93. 10.1093/icesjms/fss113 .

[6] Arrigo KR, van Dijken GL. Secular trends in Arctic Ocean net primary production. Journal of Geophysical Research: Oceans. 2011;116(C9):C09011 10.1029/2011JC007151

[7] Hunt GL, Stabeno P, Walters G, Sinclair E, Brodeur RD, Napp JM, et al Climate change and control of the southeastern Bering Sea pelagic ecosystem. Deep Sea Research Part II: Topical Studies in Oceanography. 2002;49(26):5821–53. 10.1016/S0967-0645(02)00321-1.

[8] Hunt GL, Stabeno PJ, Strom S, Napp JM. Patterns of spatial and temporal variation in the marine ecosystem of the southeastern Bering Sea, with special reference to the Pribilof Domain. Deep Sea Research Part II: Topical Studies in Oceanography. 2008;55(16–17):1919–44. 10.1016/j.dsr2.2008.04.032.

[9] Stabeno PJ, Kachel NB, Sullivan M, Whitledge TE. Variability of physical and chemical characteristics along the 70-m isobath of the southeastern Bering Sea. Deep Sea Research Part II: Topical Studies in Oceanography. 2002;49(26):5931–43. 10.1016/S0967-0645(02)00327-2.

[10] Hunt GL, Jr., Allen BM, Angliss RP, Baker T, Bond N, Buck G, et al. Status and trends of the Bering Sea region, 2003–2008. In: McKinnell SM, Dagg MJ, editors. Marine Ecosystems of the North Pacific Ocean, 2003–2008 PICES Special Publication 4, 393 p2010. p. 196–267.

[11] Lomas MW, Moran SB, Casey JR, Bell DW, Tiahlo M, Whitefield J, et al Spatial and seasonal variability of primary production on the Eastern Bering Sea shelf. Deep Sea Research Part II: Topical Studies in Oceanography. 2012;65–70(0):126–40. 10.1016/j.dsr2.2012.02.010.

[12] Hunt GL, Coyle KO, Eisner LB, Farley EV, Heintz RA, Mueter F, et al Climate impacts on eastern Bering Sea food webs: a synthesis of new data and an assessment of the Oscillating Control Hypothesis. ICES Journal of Marine Science: Journal du Conseil. 2011;68(6):1230–43. 10.1093/icesjms/fsr036

[13] Sigler MF, Stabeno PJ, Eisner LB, Napp JM, Mueter FJ. Spring and fall phytoplankton blooms in a productive subarctic ecosystem, the eastern Bering Sea, during 1995–2011. Deep Sea Research II. 2014;109:71–83. 10.1016/j.dsr2.2013.12.007

[14] Stauffer BA, Goes JI, McKee K, do Rosario Gomes H, Stabeno P. Comparison of spring time phytoplankton community composition in two cold years from the western Gulf of Alaska into the southeastern Bering Sea. Deep Sea Research II. 2014;109:57–70.

[15] Coyle KO, Pinchuk AI. The abundance and distribution of euphausiids and zero-age pollock on the inner shelf of the southeast Bering Sea near the Inner Front in 1997–1999. Deep Sea Research II. 2002;49:6009–30.

[16] Coyle KO, Pinchuk AI, Eisner LB, Napp JM. Zooplankton species composition, abundance and biomass on the eastern Bering Sea shelf during summer: The potential role of water-column stability and nutrients in structuring the zooplankton community. Deep Sea Research II. 2008;55:1775–91.

[17] Stabeno P, Napp J, Mordy C, Whitledge T. Factors influencing physical structure and lower trophic levels of the eastern Bering Sea shelf in 2005: Sea ice, tides and winds. Progress in Oceanography. 2010;85(3–4):180–96. 10.1016/j.pocean.2010.02.010.

[18] Emery WJ, Thomson RE. Data Analysis Methods in Physical Oceanography Amsterdam: Elsevier Science B.V.; 2001. 638 p.

[19] Brierley AS, Saunders RA, Bone DG, Murphy EJ, Enderlein P, Conti SG, et al Use of moored acoustic instruments to measure short-term variability in abundance of Antarctic krill. Limnology and Oceanography: Methods. 2006;4:18–29.

[20] Kunze E, Dower J.F., Beveridge I., Dewey R., Bartlett K.P. Observations of Biologically Generated Turbulence in a Coastal Inlet. Science. 2006;22:1768–70. 10.1126/science.1129378 16990545

[21] Fofonoff P, Millard RCJ. Algorithms for computation of fundamental properties of seawater UNESCO, 1983.

[22] Codiga DL. Density stratification in an estuary with complex geometry: Driving processes and relationship to hypoxia on monthly to inter-annual timescales. Journal of Geophysical Research: Oceans. 2012;117(C12):C12004 10.1029/2012JC008473

[23] Davis P. Interpolation and Approximation University of Michigan: Dover Publications; 1975. 393 p.

[24] Little EM, Allen MB, Wright FF. Field Measurement of Light Penetration through Sea Ice. Arctic. 1972;25(1):28–33.

[25] Cetinić I, Toro-Farmer G, Ragan M, Oberg C, Jones BH. Calibration procedure for Slocum glider deployed optical instruments Optics Express. 2009;17(18):15420–30. 10.1364/OE.17.015420 19724540

[26] Earp A, Hanson CE, Ralph PJ, Brando VE, Allen S, Baird M, et al Review of fluorescent standards for calibration of in situ fluorometers: Recommendations applied in coastal and ocean observing programs. Optics Express. 2011;19(27):26768–82. 10.1364/OE.19.026768 22274260

[27] Owen RW. Microscale and finescale variations of small plankton in coastal and pelagic environments. Journal of Marine Research. 1989;47(1):197–240.

[28] Dekshenieks MM, Donaghay PL, Sullivan JM, Rines JEB, Osborn TR, Twardowski MS. Temporal and spatial occurrence of thin phytoplankton layers in relation to physical processes. Marine Ecology Progress Series. 2001;223:61–71.

[29] Lorenzen CJ. A method for the continuous measurement of in vivo chlorophyll concentration. Deep-Sea Research. 1966;13:223–7.

[30] Watkins JL, Brierley AS. Verification of acoustic techniques used to identify and size Antarctic krill. ICES Journal of Marine Science. 2002;59:1326–36.

[31] Reiss CR, Cossio AM, Loeb VL, Demer DA. Variations in the biomass of Antarctic krill (*Euphausia superba*) around the South Shetland Islands, 1996–2006. ICES Journal of Marine Science. 2008;65(4):497–508.

[32] DeRobertis A, McKelvey DR, Ressler PH. Development and application of an empirical multifrequency method for backscatter classification. Canadian Journal of Fisheries and Aquatic Sciences. 2010;67:1459–74.

[33] Smith PE, Flerx W, Hewitt. RP. The CalCOFI vertical egg tow (CalVET) net. 1985; In: Lasker R, editor. An Egg Production Method for Estimating Spawning Biomass of Pelagic Fish: Application to the Northern Anchovy, *Engraulis mordax*, NOAA Technical Report NMFS 36; 1985. p. 27–32.

[34] Demer DA, Conti SG. Validation of the stochastic distorted-wave Born approximation model with broad bandwidth total target strength measurements of Antarctic krill. ICES Journal of Marine Science. 2003;60:625–35.

[35] Miksis-Olds JL, Stabeno PJ, Napp JM, Pinchuk AI, Nystuen JA, Warren JD, et al Ecosystem response to a temporary sea ice retreat in the Bering Sea: Winter 2009. Progress in Oceanography. 2013;111(0):38–51. 10.1016/j.pocean.2012.10.010.

[36] Gardner GA, Szabo, I. British Columbia pelagic marine Copepoda: An identification manual and annotated bibliography. In: Canadian Special Publication of Fisheries Aquatic Sciences. 62; 1982. p. 536.

[37] Stanton TK. From acoustic scattering models of zooplankton to acoustic surveys of large regions. Proceedings of the IEEE Colloquium on Recent Advances in Sonar Applied to Biological Oceanography. London, UK; 1998.

[38] Clarke KR, Warwick RM. Change in marine communities: an approach to statistical analysis and interpretation 2nd edition. Plymouth: UK:PRIMER-E; 2001.

[39] Menke W, Menke J. Environmental Data Analysis with Matlab Amsterdam: Elsevier; 2012. 288 p.

[40] Zhang J, Woodgate R, Mangiameli S. Towards seasonal prediction of the distribution and extent of cold bottom waters on the Bering Sea shelf. Deep Sea Research Part II: Topical Studies in Oceanography. 2012;65–70(0):58–71. 10.1016/j.dsr2.2012.02.023.

[41] Flynn KJ. Attack is not the best form of defense: Lessons from harmful algal bloom dynamics. Harmful Algae. 2008;8(1):129–39.

[42] Clough J, Strom S. Effects of *Heterosigma akashiwo* (Raphidophyceae) on protist grazers: laboratory experiments with ciliates and heterotrophic dinoflagellates. Aquatic Microbial Ecology. 2005;39:121–34.

[43] Turner JT, Ianora A, Esposito F, Carotenuto Y, Miralto A. Zooplankton feeding ecology: does a diet of *Phaeocystis* support good copepod grazing, survival, egg production and egg hatching success? Journal of Plankton Research. 2002;24(11):1185–95. 10.1093/plankt/24.11.1185

[44] Nejstgaard JC, Tang KW, Steinke M, Dutz J, Koski M, Antajan E, et al Zooplankton Grazing on *Phaeocystis*: A Quantitative Review and Future Challenges. Biogeochemistry. 2007;83(1/3):147–72. 10.2307/20456477

[45] Liu H, Hopcroft R. Growth and development of *Pseudocalanus* spp. in the northern Gulf of Alaska. Journal of Plankton Research. 2008;30(8):923–35.

[46] Coyle KO, Eisner LB, Mueter FJ, Pinchuk AI, Janout MA, Cieciel KD, et al Climate change in the southeastern Bering Sea: impacts on pollock stocks and implications for the oscillating control hypothesis. Fisheries Oceanography. 2011;20(2):139–56.

[47] Baier CT, Napp JM. Climate-induced variability in *Calanus marshallae* populations. Journal of Plankton Research. 2003;25(7):771–82.

[48] Stabeno PJ, Farley EV Jr, Kachel NB, Moore S, Mordy CW, Napp JM, et al A comparison of the physics of the northern and southern shelves of the eastern Bering Sea and some implications for the ecosystem. Deep Sea Research Part II: Topical Studies in Oceanography. 2012;65–70(0):14–30. 10.1016/j.dsr2.2012.02.019.

[49] Baier CT, Terazaki M. Interannual variability in a predator–prey interaction: climate, chaetognaths and copepods in the southeastern Bering Sea. Journal of Plankton Research. 2005;27(11):1113–25. 10.1093/plankt/fbi078

[50] Nakagawal Y, Endo Y, Taki K. Contributions of heterotrophic and autotrophic prey to the diet of euphausiid, *Euphausia pacifica* in the coastal waters off northeastern Japan. Polar Bioscience. 2002;15:52–65.

[51] Atkinson A, Snÿder R. Krill-copepod interactions at South Georgia, Antarctica, I. Omnivory by *Euphausia superba*. 160 1997;(63–76).

[52] Aydin K, Mueter F. The Bering Sea—A dynamic food web perspective. Deep Sea Research II. 2007;54:2501–25.

